# Anterior Cruciate Ligament Reconstruction With Quadriceps Tendon Autograft: Surgical Technique Using Augmentation With a Biocomposite Scaffold

**DOI:** 10.1016/j.eats.2023.08.003

**Published:** 2023-11-27

**Authors:** Kaitlin Pyrz, Audria Wood, Collier Campbell, Eugene Brabston, Thomas Evely, Aaron Casp, Amit Momaya

**Affiliations:** a Augusta University/University of Georgia Medical Partnership, Athens, Georgia, U.S.A.; b Department of Orthopaedics, University of Alabama at Birmingham, Birmingham, Alabama, U.S.A.

## Abstract

Anterior cruciate ligament (ACL) reconstruction augmentation continues to be widely studied. Both biologic and synthetic augments have been employed to enhance ACL healing and provide early protection. The BioBrace is a biocomposite scaffold that both mechanically reinforces the graft while biologically enhancing graft healing. The purpose of this article is to describe augmentation of an ACL reconstruction with BioBrace.

The incidence of anterior cruciate ligament (ACL) tears continues to remain high in the United States. Although there are several graft options, the quadriceps tendon graft is more widely used due to its favorable biomechanics, low donor-site morbidity, large cross-sectional area, and graft versatility.^1^^,^^2^ Graft failure rate ranges from 3% to 14% in general.^3^ Such failure can be from a variety of reasons, including technical, traumatic, or biologic.^4^, ^5^, ^6^ The factors surrounding biologic failure continue to remain elusive.

Many surgeons augment the graft with an internal brace.^7^ Although adding a purely structural support does improve early mechanical properties, it does not appear to increase the collagen content of the graft nor support graft integration or biologic healing.

The BioBrace (Biorez, New Haven, CT) is a biocomposite scaffold that, when sutured onto the graft, mechanically reinforces the ligament and may act to accelerate the healing process of the graft. The BioBrace is composed of bioresorbable poly (l-lactide) microfilaments and highly porous type I collagen. Its biomechanical properties enable it to share loads and assist in the biologic healing of ligaments, gradually resorbing as the tissue remodels. We describe our method for graft preparation and ACL reconstruction using an all-inside quadricep tendon autograft augmented with BioBrace.

## Surgical Technique (With Video Illustration)

Each stage of the surgical technique is demonstrated in Video 1.

### Graft Harvest

**Fig 1:**
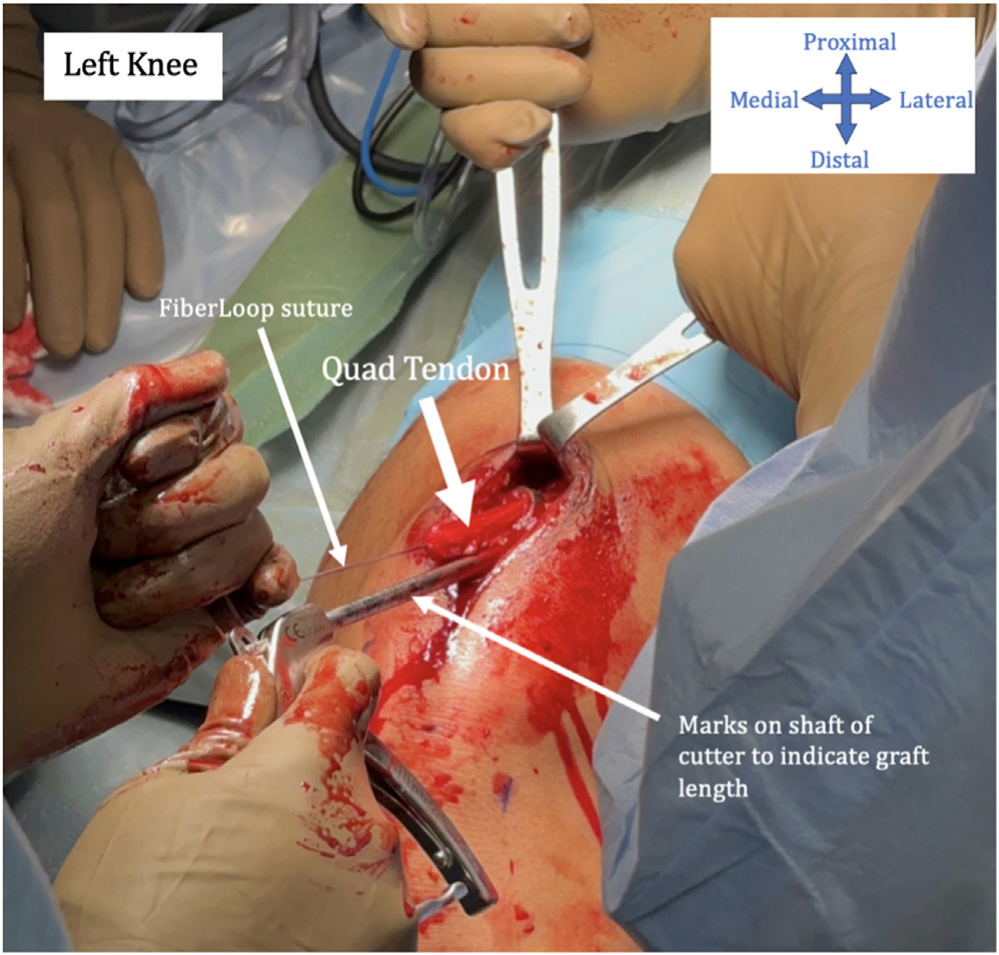
The quadriceps tendon is harvested in a standard fashion using a cigar cutter (Arthrex, Naples, FL). Aim to achieve an approximately 70-mm graft length. This is a left knee.

The quadriceps tendon is harvested in standard fashion with the aim to achieve an approximately 70-mm graft length. This is shown in Figure 1. The graft is then taken to the back table for preparation.

### BioBrace Preparation and Graft Tensioning

**Fig 2:**
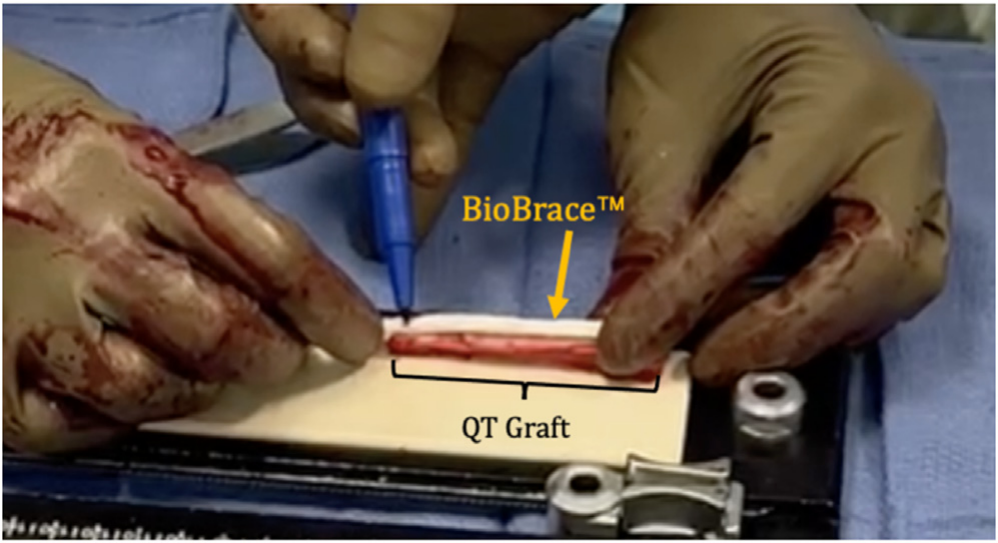
The BioBrace strip is placed on top of the graft. A skin marker approximates the cut so that the length of the BioBrace matches the length of the graft. A cut is then made according to that mark (not pictured).

**Fig 3:**
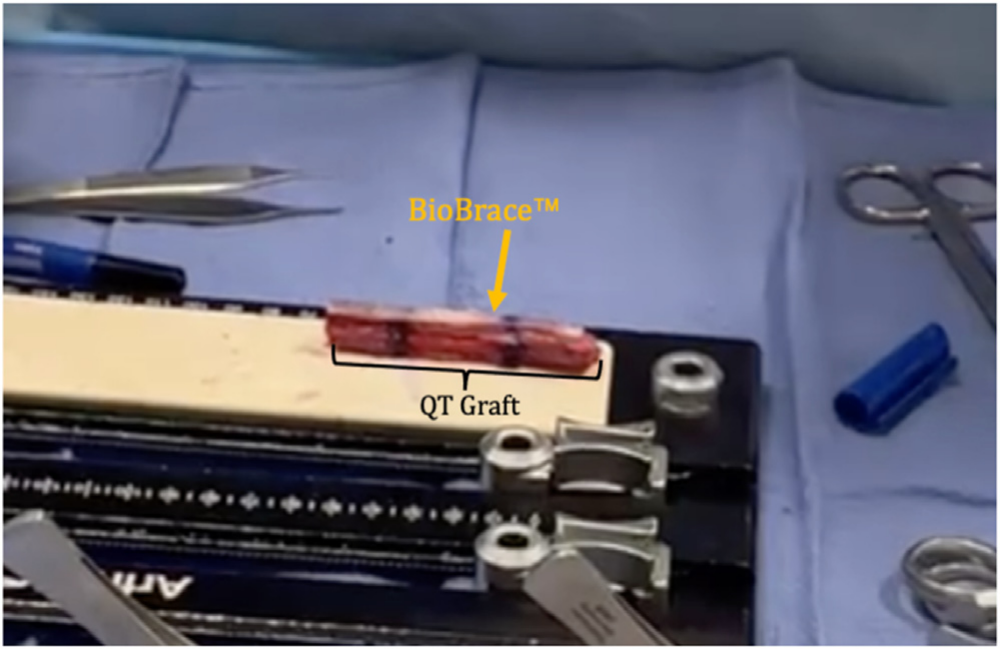
A skin marker is used to create circumferential lines around the graft approximately 20 mm from each end, and the corresponding marks are made on the BioBrace.

The BioBrace, which measures 5 mm in thickness, is measured and cut to match the length of the quadriceps tendon graft itself (Fig 2). The BioBrace is then hydrated with a saline solution to make it more pliable during preparation. The graft is marked at 20 mm from each end, identifying the portions that will be within the femoral and tibial tunnels (Fig 3).

**Fig 4:**
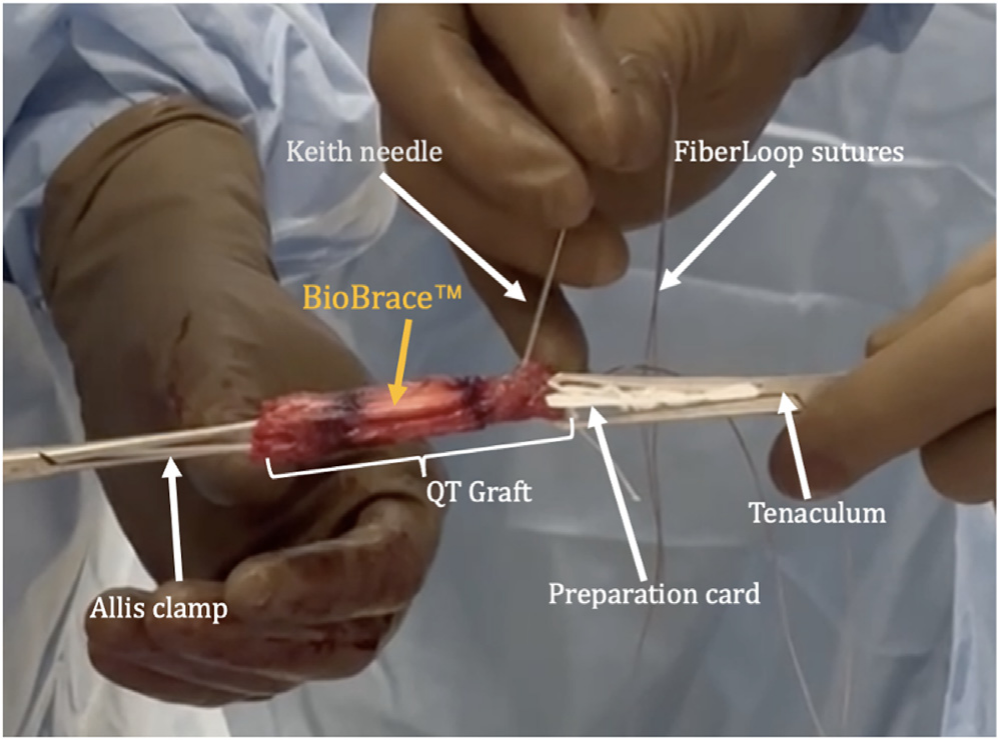
The graft and BioBrace are held together with an Allis clamp on one side and a tenaculum on other while the sutures are passed through using a Keith needle. Be sure to pierce all structures (e.g., both the graft and the BioBrace) in each pass.

The FiberTag TightRope Quad ACL graft prep system (Arthrex, Naples, FL) is used to prepare the graft. The graft and BioBrace are clamped together to the preparation card with an Allis clamp and the included tenaculum. The FiberLoop stitches are passed through the FiberTag in the recommended fashion using the Keith needle, with care taken to pierce the tape, the quadriceps graft, as well as the BioBrace with each pass (Fig 4). Starting 20 mm from the end of the graft, 2 passes are made toward the end of the graft. Then, the needle is passed through the slit in the card, and a few additional passes are made, ensuring that the BioBrace is secured to the autograft. The ends of the suture are tied, and the knot buried within the graft. This process is repeated for the other end of the quadriceps graft.

**Fig 5:**
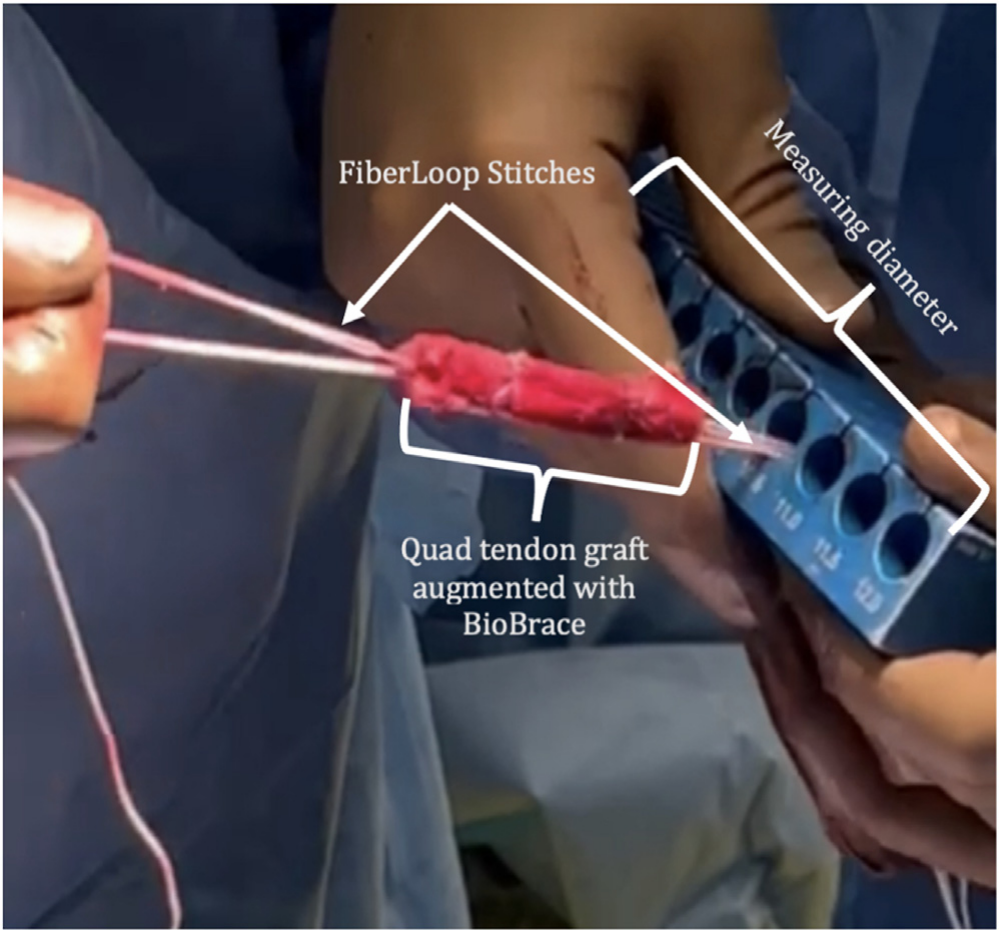
The diameter of the graft is then measured. It may be trimmed as needed until the desired diameter is achieved.

**Fig 6:**
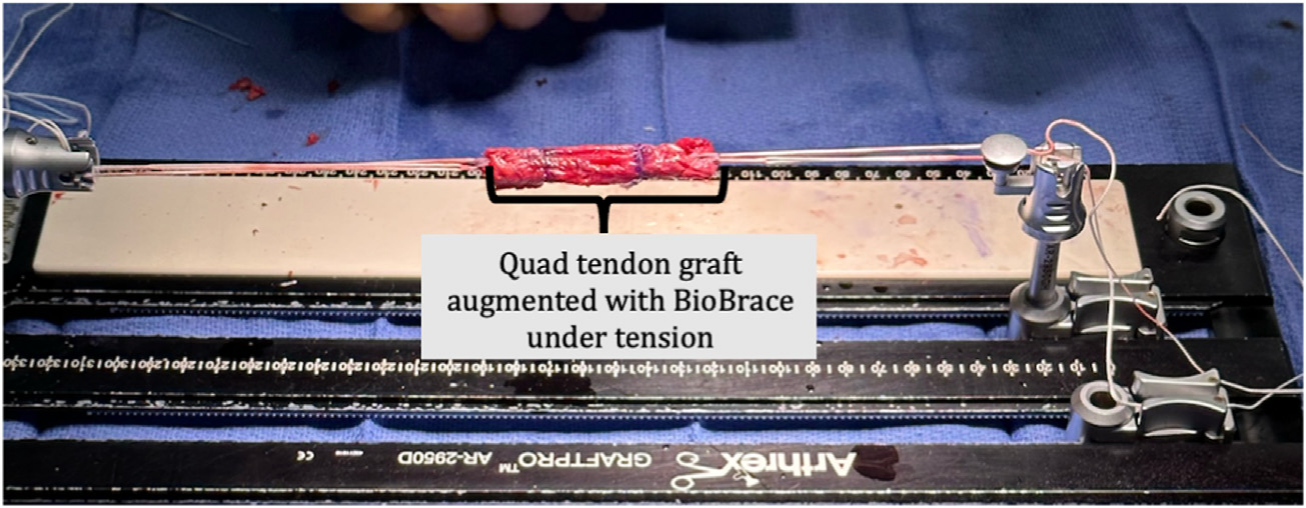
The graft is placed on the graft prep station and tensioned.

The diameter of the graft is then measured (Fig 5), and the graft is then placed on the graft prep station and tensioned within a compression tube to ensure it remains the desired diameter (Fig 6).

### ACL Reconstruction

**Fig 7:**
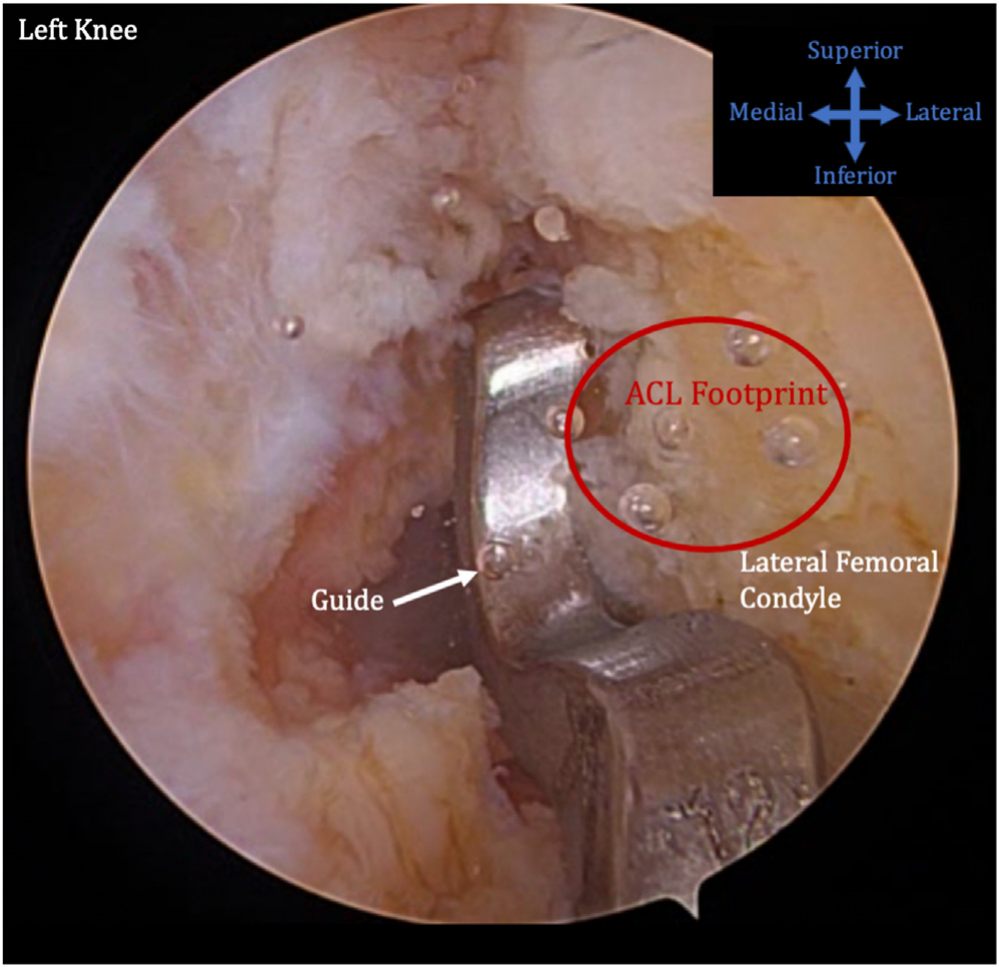
Pictured is the view through the anteromedial portal of the left knee with the patient supine and knee flexed to approximately 90°. The guide is positioned at the anatomic location on the lateral wall of the intercondylar notch. (ACL, anterior cruciate ligament.)

**Fig 8:**
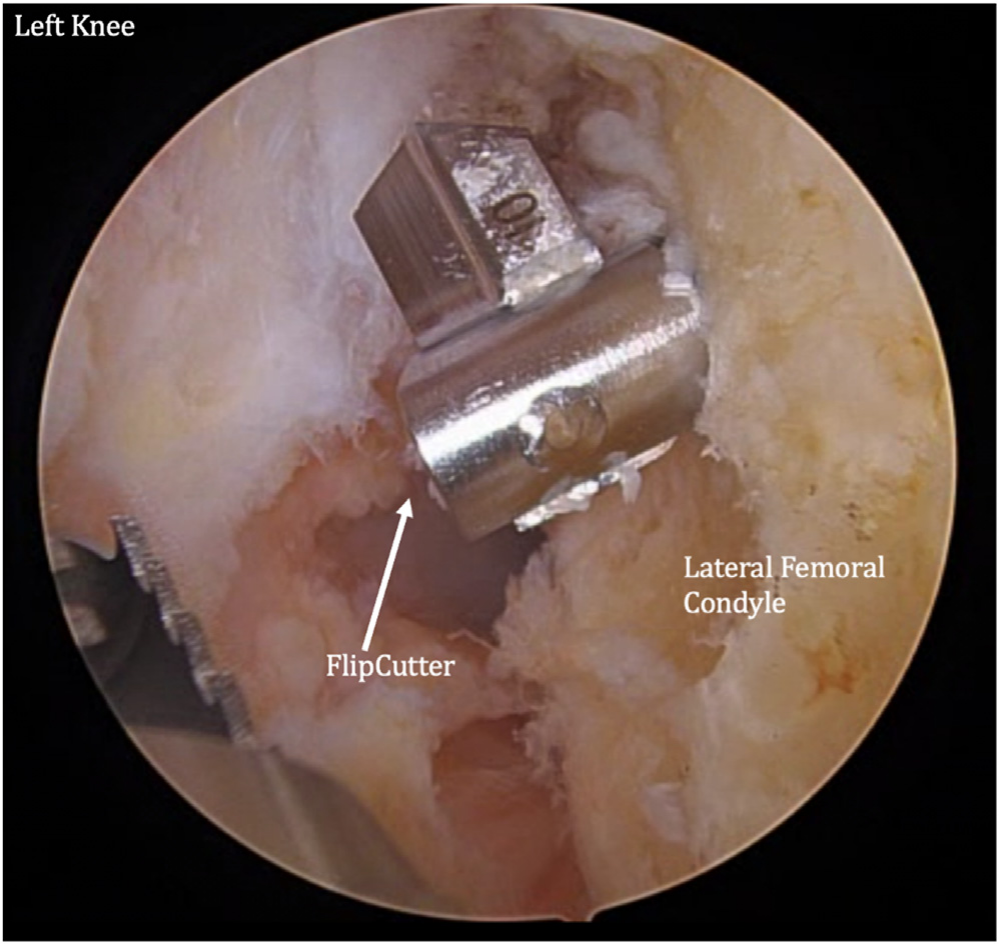
A FlipCutter is used to drill from outside-in. The FlipCutter is then used to ream to the appropriate diameter and depth. A goal of approximately 25 mm length is targeted. Pictured is the viewing from the anteromedial portal of the left knee at 90° flexion. Shown is the view of the (anteromedial) portal on the left knee at 90° flextion while the patient is supine. A passing stitch is then passed into the femoral socket.

**Fig 9:**
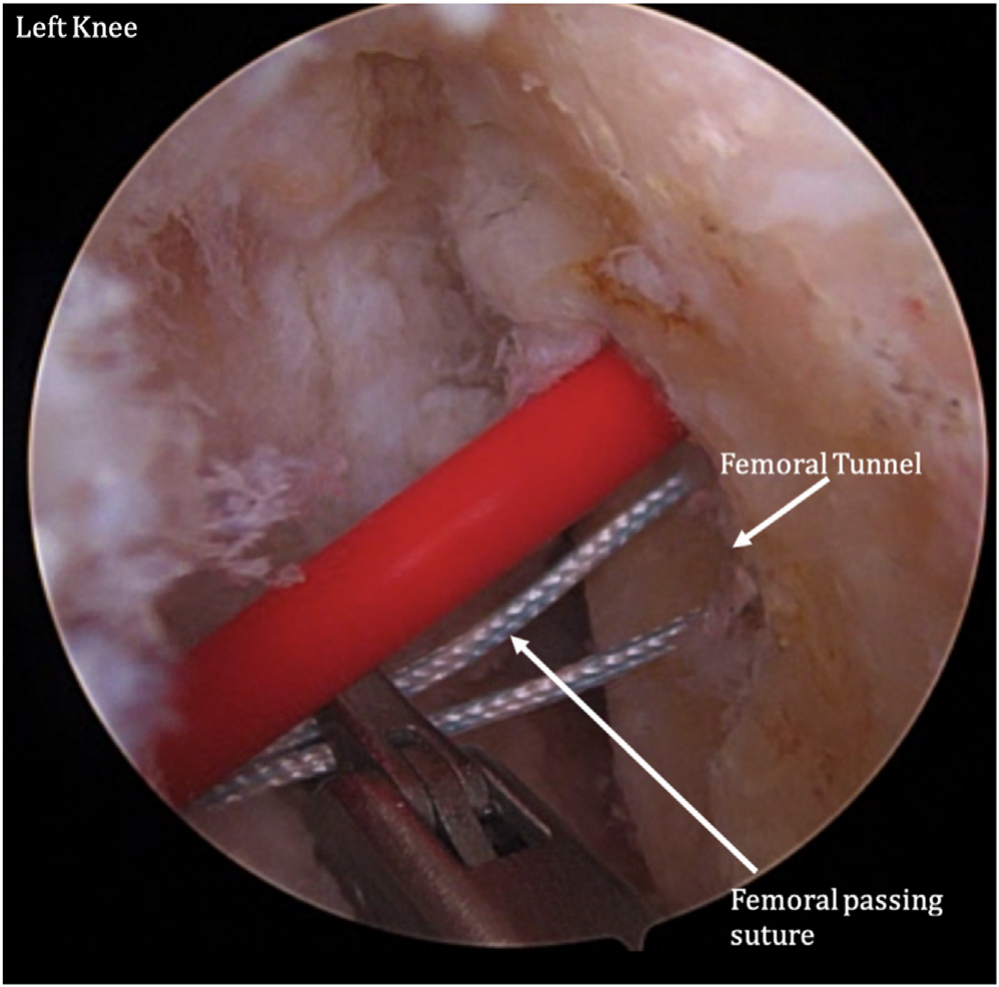
Shown is the view of the through the anteromedial portal of the left knee at 90° flexion while the patient is supine. A passing stitch is then passed into the femoral socket.

**Fig 10:**
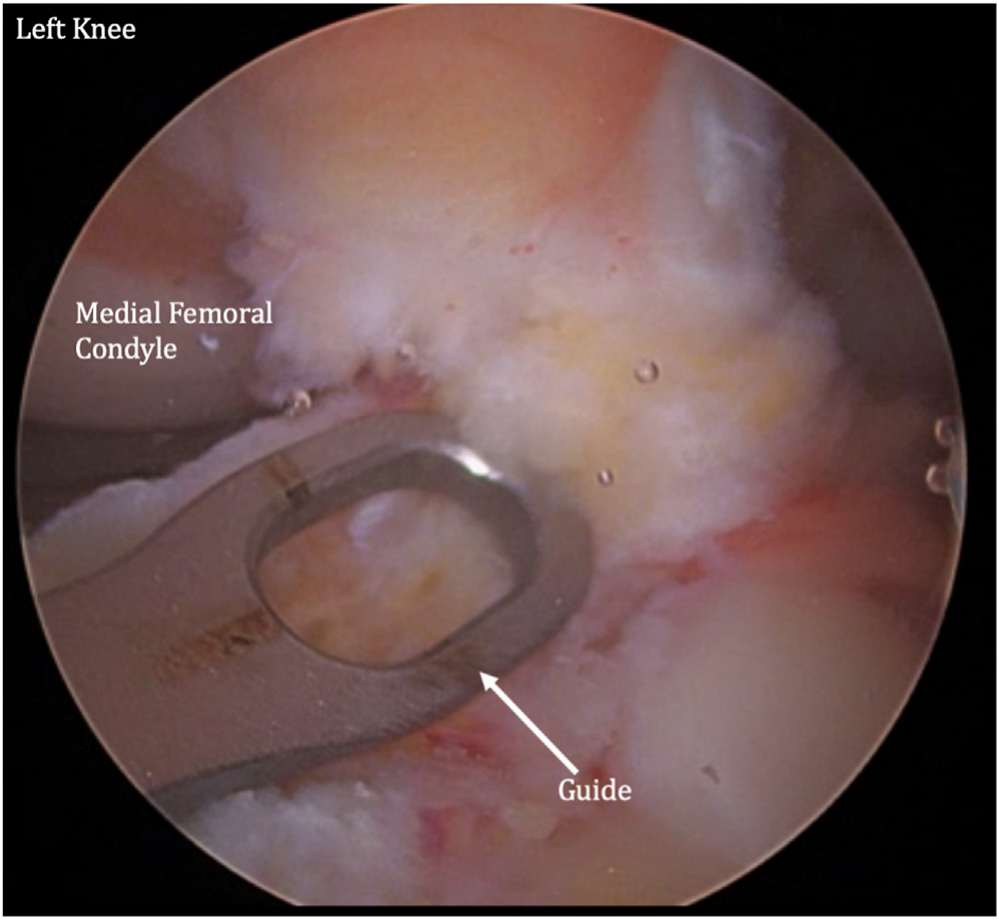
View is through the anterolateral portal of the the left knee at 90° flexion. A tunnel is then drilled through the tibial footprint in similar fashion. First, the drill guide is placed on the anterior tibial cortex.

**Fig 11:**
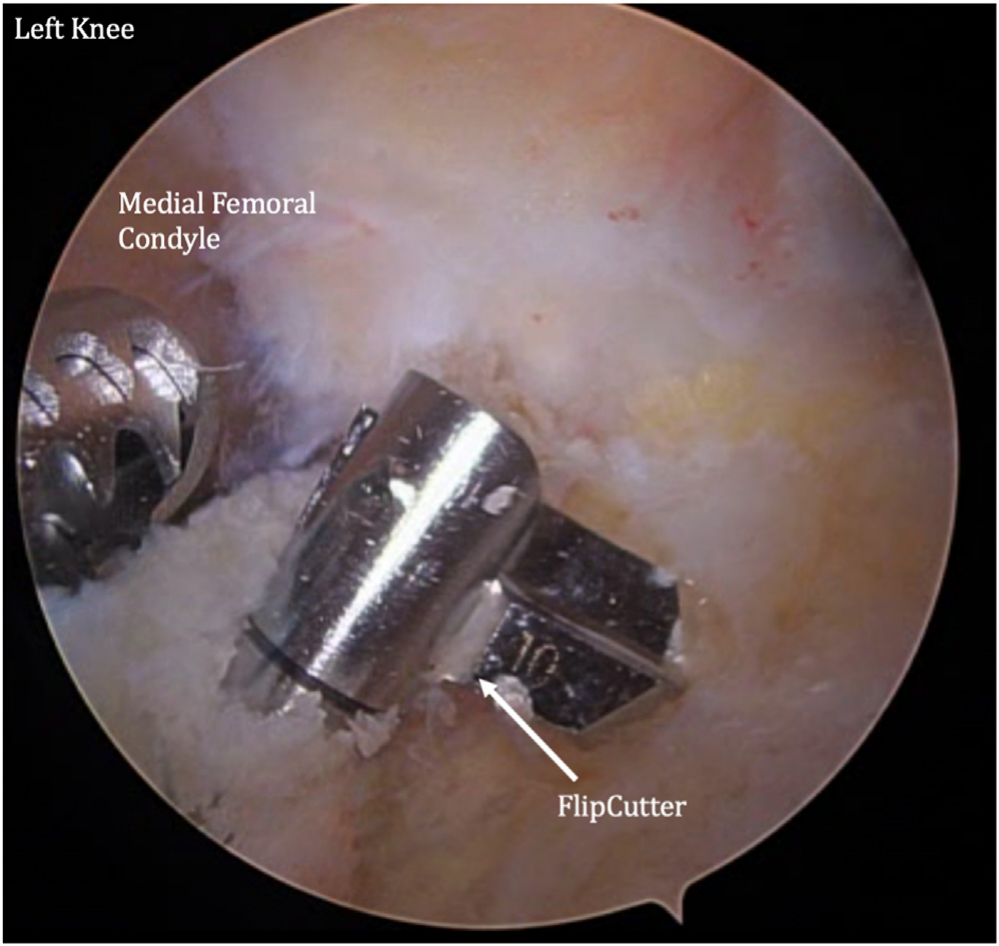
Viewing through an anterolateral portal of the left knee of supine patient with the knee flexed to approximately 90°. The FlipCutter is drilled into the tibial footprint of the ACL and then drilled out the appropriate depth. A goal of approximately 25 mm length is targeted. (ACL, anterior cruciate ligament.)

The femur and tibia are prepared for ACL reconstruction by removal of remnant ACL, and the guide is positioned at the anatomic location on the lateral wall of the intercondylar notch (Fig 7). A FlipCutter (Arthrex) is used to drill from outside-in. The FlipCutter is then used to ream to the appropriate diameter and depth (Fig 8), and a passing stitch is then passed into the femoral socket (Fig 9). Similarly, the tibial guide is placed through the medial portal, and the drill guide is then placed on the anterior tibial cortex (Fig 10). The FlipCutter is drilled into the tibial footprint of the ACL and then drilled to the appropriate depth, and a passing suture is placed (Fig 11). A goal of approximately 25 mm length is targeted for each of these sockets.

### Graft Passage

**Fig 12:**
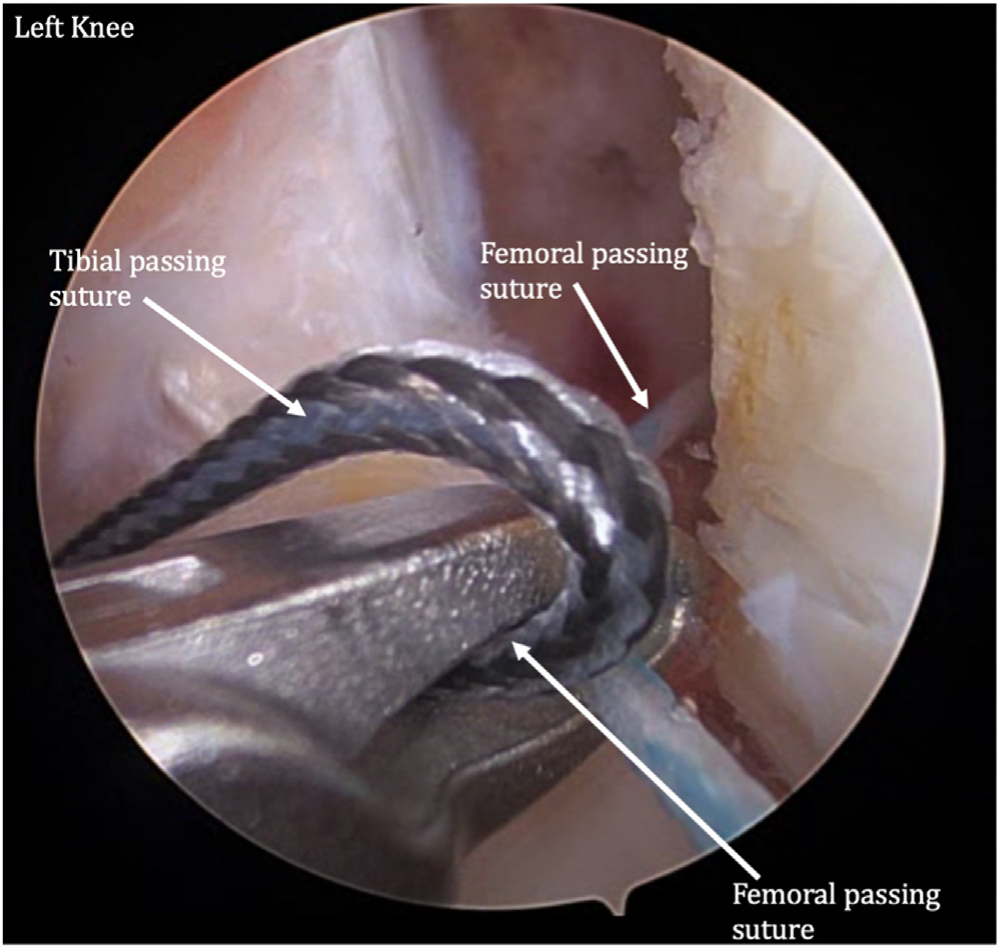
A tibial passing suture is placed. The femoral and tibial passing sutures are pulled out through the medial portal in one pass.

**Fig 13:**
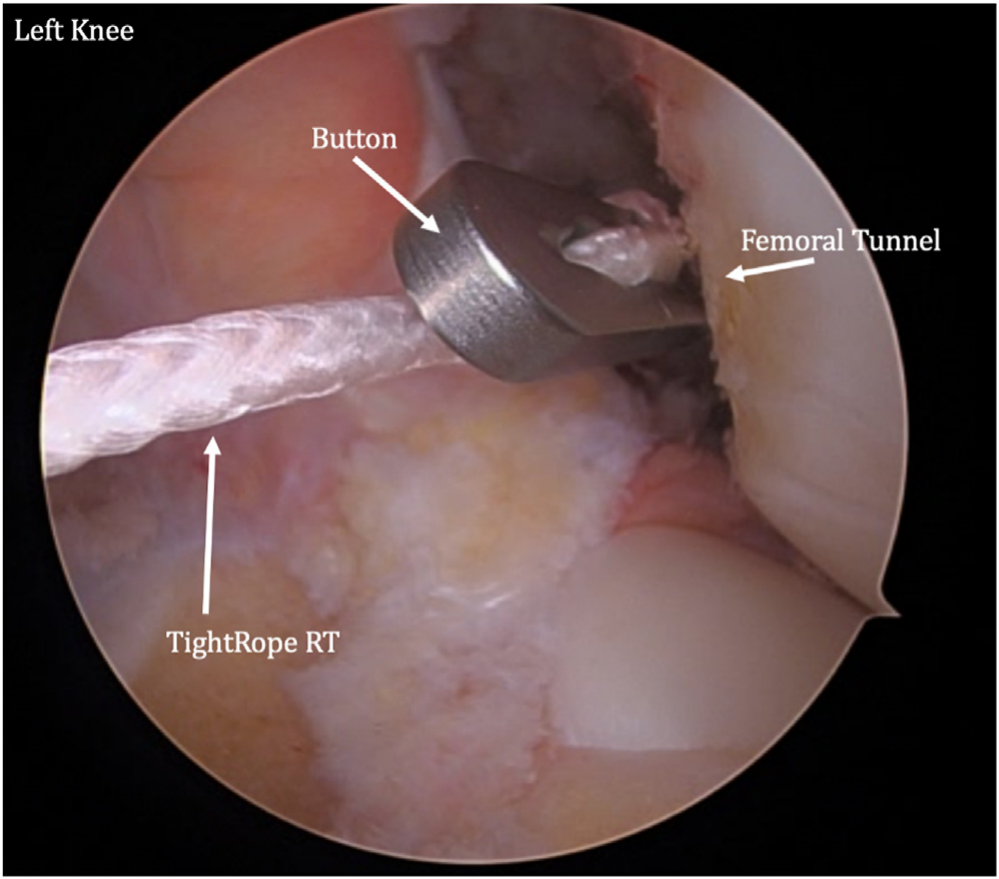
The graft is then shuttled into the knee through the medial portal. The femoral button is pulled through the femoral socket and then flipped so it rests flush on the femoral cortex.

**Fig 14:**
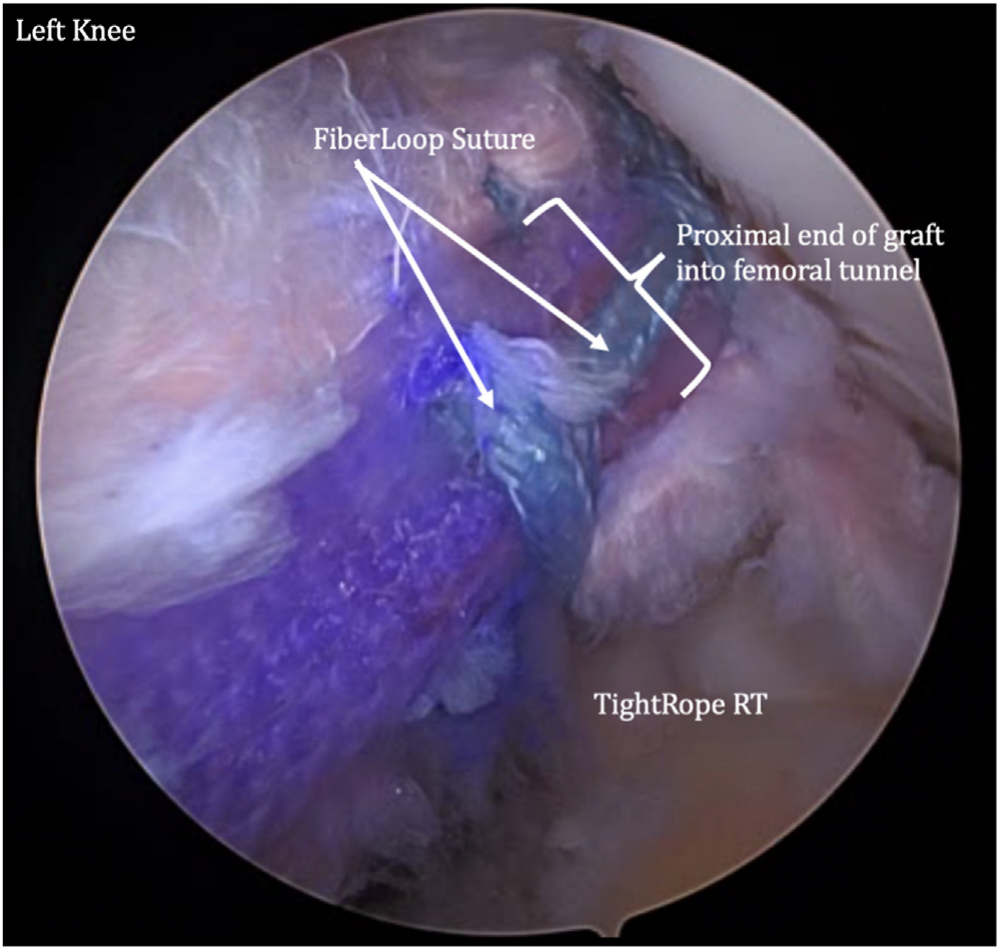
The femoral side of the graft is pulled into the socket approximately 20 mm.

**Fig 15:**
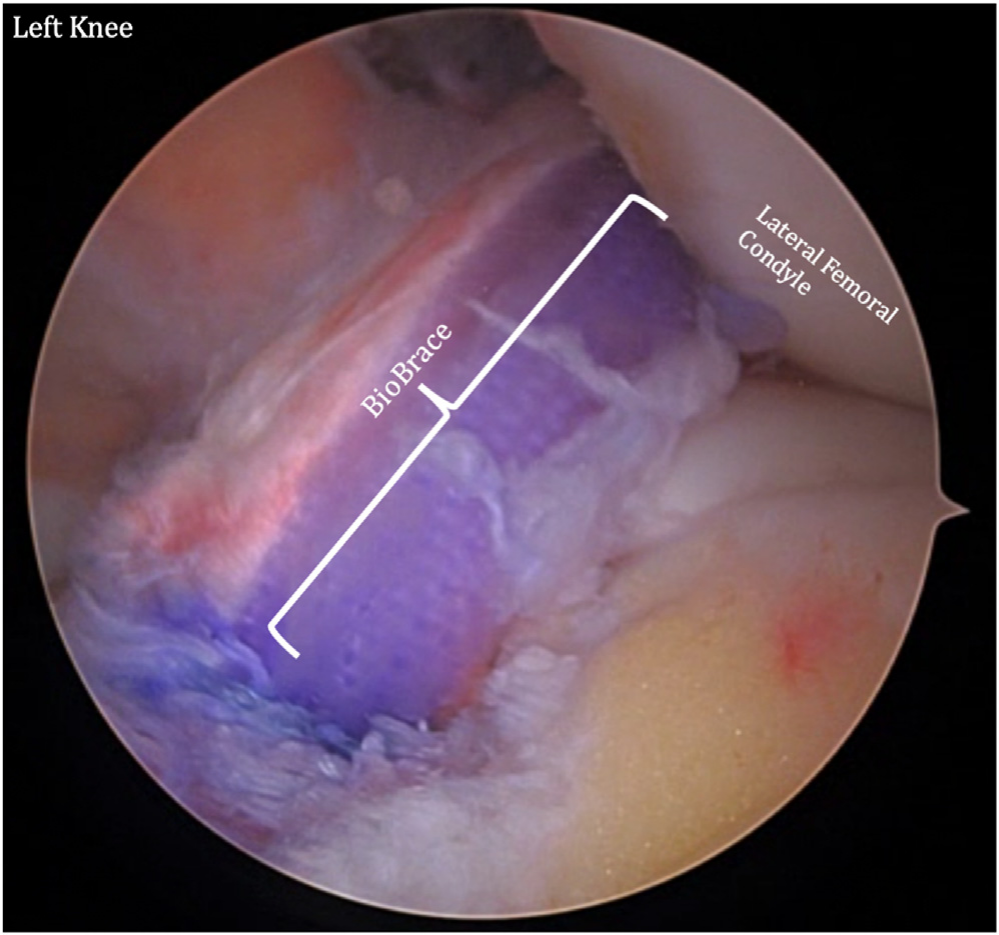
The tibial-sided sutures are pulled out the tibial tunnel, and the graft is docked into the tibial socket.

The femoral and tibial passing sutures are pulled out through the medial portal, with care taken to avoid any soft-tissue bridge (Fig 12). The femoral button is pulled up through the femoral socket and then flipped so it rests flush on the femoral cortex (Fig 13). The femoral side of the graft is pulled into the socket approximately 20 mm (Fig 14). The tibial-sided sutures are pulled out the tibial tunnel, and the graft is docked into the tibial socket (Fig 15).

Tibial fixation is performed using a button on the anteromedial tibial cortex, and the tibial tightrope sutures are shortened until fully seated. After cycling the knee, final tension is applied to the TightRope on both the femoral and tibial sides, and the TightRope sutures are tied with square knots to fully set the fixation. At this point, the augmented graft should be secured within the knee. Postoperative rehabilitation is implemented in typical fashion for ACL reconstruction, without any alterations due to the use of the BioBrace.

## Discussion

This Technical Note describes the use of a biocomposite scaffold implant to augment an autograft ACL reconstruction. The impetus behind augmentation rests on the idea that such it may enhance the remodeling and biologic integration of the graft in addition to providing some early mechanical strength to the graft.

The concept of bovine scaffold augmentation has been used in other areas of the body, including the medial collateral ligament,^8^ distal biceps tendon,^9^ and rotator cuff.^10^ The BioBrace has been studied on a basic science level in large animal models.^8^^,^^10^ In addition to providing strength, it rapidly incorporates and ultimately resorbs while the native tissue remains.^10^^,^^11^

ACL augmentation with biologics has gained a significant amount of interest recently, including platelet-rich plasma (PRP),^2^ bone marrow aspirate concentrate (BMAC),^4^ and amnion.^5^ With regard to PRP, Radice et al.^12^ showed that the addition of PRP to ACL grafts may reduce the average time to achieve normal magnetic resonance imaging signal intensity. Forsythe et al.,^13^ in a randomized controlled trial, demonstrated that BMAC may accelerate allograft ligamentization. However, BMAC harvest may lead to some donor-site morbidity. Although the aforementioned biologics are promising, these biologics fail to provide any enhanced strength to the graft at time zero.

There has also been an interest in using synthetic material as an internal brace for ACL augmentation. A biomechanical study by Noonan et al.^14^ demonstrated that suture tape reinforcement may protect the graft from lengthening during maturation and remodeling phases of healing. Bodendorfer et al.^15^ published a matched comparative study for ACL reconstruction with hamstring grafts with or without suture augmentation. Those with suture augmentation exhibited improved patient-reported outcomes, less pain, and improved return to preinjury activity level. Although the internal brace provides mechanical strength, it fails to directly enhance the biology of the graft. In contrast, the BioBrace offers approximately 140 N of tensile strength (per Biorez, internal data) in addition to enhanced biological properties.

Our hope is that BioBrace augmentation of ACL grafts will enhance both the biology of the graft in addition to providing some strength early on during its healing and revascularization process. The advantages and disadvantages are listed in Table 1, whereas the pearls and pitfalls are listed in Table 2.

**Table 1:** Advantages and Disadvantages

Advantages	Disadvantage
Ease of technique reproducibility	Increased cost
Utility among all types of grafts (both autograft and allograft)	Increased time for graft preparation
Increased biomechanical strength at time zero	Low risk of inflammatory response to poly (l-lactide) microfilaments within BioBrace
Possibility of enhancing the anterior cruciate ligament graft biology with maturation, collagen thickness, and graft incorporation	Possibility of increased infection risk
Ability to augment slightly smaller diameter grafts	Lack of long-term clinical outcomes
No donor-site morbidity in comparison with bone marrow aspiration concentrate

**Table 2:** Pearls and Pitfalls

Pearls	Pitfalls
Ensure appropriate length of BioBrace to match graft length	Avoid doubling over a BioBrace on a thicker graft in order to avoid arthrofibrosis or overstuffing of the notch
Ensure appropriate hydration of the BioBrace in either saline, platelet-rich plasma or bone marrow aspirate concentrate	
During graft preparation, be careful to pierce the BioBrace on each pass of the Keith needle to ensure appropriate fixation	

## Supplementary Data

ICMJE author disclosure forms

Video 1 This video shows an ACL reconstruction using an all-inside quadricep tendon autograft augmented with BioBrace. BioBrace is a biocomposite scaffold for mechanical and biologic enhancement. The graft harvesting and reconstruction follows standard procedure. The video shows greater attention to the BioBrace-specific steps. (ACL, anterior cruciate ligament.)
